# Outcomes and Complications of Sutured Scleral-Fixated Foldable Intraocular Lens Implantation: A Retrospective Study of 5-Year Follow-Up

**DOI:** 10.1155/2021/5525064

**Published:** 2021-07-16

**Authors:** Ting Yu, Mengting Yu, Wenjie Wu, Xinna Wu, Suzhen Xiao, Jialiang Mao, Yanling Wang

**Affiliations:** ^1^Ophthalmology Department, Provincial Clinical Medical College of Fujian Medical University, Fuzhou 350001, China; ^2^Ophthalmology Department, Fujian Provincial Hospital, Fuzhou 350001, China; ^3^Ophthalmology Department, Guangze County Hospital, Nanping 353000, China; ^4^Ophthalmology Department, Xiapu Funing County Hospital, Ningde 352000, China

## Abstract

**Purpose:**

To evaluate long-term outcomes and complications of sutured scleral-fixated foldable intraocular lens (IOL) implantation.

**Design:**

Retrospective study.

**Methods:**

Patients who underwent sutured scleral-fixated foldable IOL implantation using 10-0 polypropylene suture were followed up for at least 5 years at one Chinese tertiary hospital and two primary hospitals.

**Results:**

52 eyes among 48 patients (35 male and 13 female) were evaluated. The mean age (years) was 50.27 ± 20.08 (range: 6 to 81). The mean postoperative follow-up time (months) was 79.70 ± 18.84 (range: 60 to 121). The mean best-corrected visual acuity (BCVA) improved from 0.83 ± 0.69 logarithm of the minimum angle of resolution (logMAR) at baseline to 0.50 ± 0.45 logMAR at the last follow-up visit. There was improved or unchanged BCVA in 44 eyes (84.62%) and reduced BCVA in 8 eyes (15.38%). Mild intraoperative intravitreal hemorrhage was observed in 3 eyes (5.77%). Early postoperative complications included transient elevated intraocular pressure (IOP) in 5 eyes (9.62%) and hypotony in 1 eye (1.92%). Secondary epimacular membrane occurred in 5 eyes (9.62%) and retinal detachment (RD; 3 years postsurgery), subconjunctival suture knot exposure (5 years postsurgery), and persistent elevated IOP (in a GRAVES patient) occurred in 1 eye (1.92%) each. No suture erosion or breakage nor IOL dislocation was observed. No visually threatening IOL tilt or decentration was reported in any patient.

**Conclusion:**

Sutured scleral-fixated foldable IOL implantation demonstrated satisfactory long-term outcomes and rare suture-related complications. This technology was safe and did not require complicated equipment and is of considerable interest in the setting of aphakia without adequate capsule support.

## 1. Introduction

There are several mainstream surgical approaches to correct aphakia without adequate capsular support. Current choices include implantation of an iris-fixated intraocular lens (IOL) (pre- or retropupillary), sutureless intrascleral posterior chamber IOL fixation, and scleral-fixated IOL [1–8]. Although angle-supported anterior chamber IOL (AC-IOL) was adopted in 1952, its use today is limited due to high long-term risks of bullous keratopathy and glaucoma [5, 6]. Scleral-fixated IOL (SF-IOL), including sutureless and sutured fixated IOL (SSF-IOL), continues to gain acceptance among surgeons [4, 9].

Sutureless ciliary sulcus-fixation technique, as proposed by Gabor and Pavlidis [2] in 2007, attempted to avoid suture-related complications. However, most reports of implantation of sutureless intrascleral posterior chamber IOL had short (1–55 months) follow-up periods [4, 9–11] and subconjunctival IOL haptic exposure, IOL dislocation (as early as 1 day postsurgery), and pupil capture have been reported [10, 12, 13]. Additionally, the need for complicated equipment/instruments and specialized surgical skills challenge the use of such techniques in developing and underdeveloped countries, especially in primary hospitals and eye centers.

Considering these obstacles, SSF-IOL implantation remains an effective procedure. Its long-term outcomes and safety profile have been widely reported [14–24]. The most concerning late complication was IOL dislocation due to suture breakage, occurring on average at approximately 50 months postsurgery, with differences between studies and ethnic groups [15–17]. For example, suture breakage in Caucasians varied from 0% to 57.69% [21–23, 25–28] at 12–294 months of follow-up, whereas in more darkly pigmented groups such as Asians and Africans, it varied from 0% to 4.65% [14, 19, 20, 29–31] at 12–180 months of follow-up. Other reported adverse events (AEs) included lens tilt, suprachoroidal or vitreous hemorrhage, retinal detachment (RD), and endophthalmitis, which varied among studies [14, 15, 18, 32].

Several studies have retrospectively evaluated long-term outcomes and complications of SSF-IOL implantation in Asian and African patients [14, 19, 20, 29–31]. Of note, reports by Kim et al. [20] and Yang and Chao [19] were based on relatively small cohorts (15 and 29 cases, respectively), and those by Zhao et al. [24] and Rogers et al. [29] were based on follow-up periods from 6 to 99 months and from 0 to 54 months, respectively. In one large retrospective review by Luk et al. [14] with follow-up ranging from 12 to 180 months, procedures were performed by four different surgeons. The study reviewed postoperative AEs, but lacked detail regarding time of occurrence. The purpose of our study was to evaluate the long-term safety, efficacy, and clinical outcomes of SSF-IOL implantation over a 5-year period in China. Special emphasis was placed on AEs including suture-related complications, IOL dislocation, hypotony, elevated intraocular pressure (IOP), and RD.

## 2. Materials and Methods

SSF-IOL implantation was performed by the same surgeon at three sites: Fujian Provincial Hospital (tertiary referral site) and Guangze County Hospital and Xiapu County Hospital (primary hospital sites). The study was conducted in compliance with the guidelines of the Declaration of Helsinki and was approved by the ethics committees of all participating hospitals.

To evaluate long-term outcomes, we conducted a retrospective investigation of patients with at least 5 years of follow-up data between December 2009 and November 2015. In total, 101 patients (27 female and 74 male) underwent surgery during this period. We excluded 6 patients who had incomplete medical records. Among the remaining 95 patients, 46 were excluded for the following: 8 died, 4 were unable to visit clinic due to disability, and 34 were lost to follow-up. Finally, we studied 52 eyes (3 in Guangze County Hospital, 2 in Xiapu County Hospital, and 47 in Fujian Provincial Hospital) among 48 patients (35 male and 13 female). Preoperative data included demographics, best-corrected visual acuity (BCVA), IOP, lens status, previous surgeries, preexisting ocular pathologies, and history of ocular trauma. Axial length (AL) was measured by using a partial coherence interferometer (IOL Master, Carl Zeiss AG, Jena, Germany) or A-scan ultrasound biometry (AL-4000 Pachymeter, Japan) prior to surgery. The refractive power of the IOL was calculated using the SRK/T formula for AL between 21 and 26 mm and the Haigis formula for AL exceeding this interval.

Visual outcomes were measured by the distance Snellen chart preoperatively and at the last clinic visit. Pre- and postoperative BCVA were measured. Snellen acuity was converted to the logarithm of minimal angle of resolution (logMAR) VA for analysis. We used a logMAR VA of 2 and 3, respectively, to represent counting fingers and hand movement vision [33]. The spherical equivalent (SE) value was calculated as the sum of the spherical power with half of the cylindrical power. The refractive prediction error (RPE) was calculated by subtracting the estimated preoperative SE from the postoperative SE. Considering that developing AL might interfere with RPE, we excluded subjects who were less than 18 years of age.

Early postoperative complications were defined as AEs occurring within 1 month postsurgery. Any AE occurring after 1 month was considered a late complication. We observed the 10-0 polypropylene suture/suture knot with a slit-lamp and, when the suture/suture knot was visible under the conjunctival, we used bulbar conjunctival fluoresce staining to determine whether the suture/suture knot was exposed beyond the conjunctival epithelium.

### 2.1. Surgical Technique

**Figure 1:**
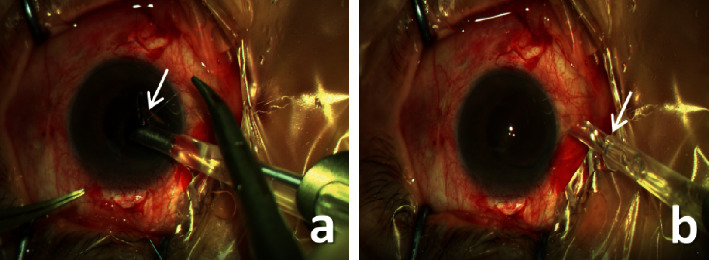
Sutured scleral-fixated implantation with a one-piece foldable IOL. (a) The suture was tied to the maximum radian of the IOL haptic with at least 5 knots. (b) After withdrawing the plunger, the haptic drew back into the cartridge.

Procedures were performed by the same surgeon using a similar technique (video and supplemental digital content are available at https://drive.google.com/file/d/1Ff5wC1Uf1pqohpw1j-S3kiiTwfMbbjhp/view?usp=sharing). Retrobulbar or general anesthesia (2 children) was administered. A three-piece (ZA9003, Tecnis, AMO) or one-piece (ZCB00, Tecnis, AMO) IOL was implanted. Two opposing limbus-based triangle scleral flaps were prepared 1.5 mm from the limbus at 4-5 o'clock and 10-11 o'clock in 48 eyes. In 4 eyes with a partial residual capsule, a single-suture scleral fixation was used to position the IOL haptic at the absent capsular position. The ab externo technique was used as described below. A straight needle carrying a 10-0 polypropylene suture was inserted into the posterior chamber through one scleral flap. A 26-gauge needle was then inserted through the opposite scleral flap to pull the straight needle out of the eye within its barrel. Then, a 2.4 or 3.0 mm corneal incision was made at 9 o'clock and the suture was pulled out through the corneal incision and cut off. A foldable IOL was loaded in the injector, part of the foregoing (leading) haptic was pushed out of the cartridge, and the suture was tied with at least 5 knots to the maximum radian of the IOL haptic to prevent suture slippage (Figure 1(a)). For the three-piece IOL (38 subjects), the haptic end was heated to create a mushroom-shaped flange of about 0.16 mm. For the one-piece IOL (10 subjects), the end was not heated, but the haptic was slightly depressed by the suture to reduce suture movement. Then, after tying the suture to the leading haptic, the plunger was withdrawn (Figure 1(b)), so that the IOL could be implanted into the posterior chamber through a smaller (2.4 mm) corneal incision. Subsequently, the cartridge was inserted into the corneal incision and the posterior (trailing) haptic was left outside the incision for suture fixation as aforementioned. Then, the sutured posterior haptic was carefully inserted into the posterior chamber through the corneal incision using microforceps. After tensioning the sutures, the IOL was placed in a central position and the corneal incision was closed using a 10-0 nylon suture followed by suturing of the scleral flaps and conjunctiva. For secondary lens implantation in eyes without coexistent vitreoretinal disorders, no vitrectomy was performed and infusion or AC maintainer was not applied. For those with minor vitreous incarceration in the pupil area/corneal incision, a scissors was used to excise it. In 4 eyes with prior pars plana vitrectomy (PPV), vitreous infusion was used to maintain intraocular stability. In 5 eyes with RD, PPV with SSF-IOL implantation was performed at the same time.

### 2.2. Statistical Analysis

Means and standard deviations (SDs) of the quantitative variables were calculated. A paired *t*-test was used to detect differences in quantitative variables when data obeyed normal distribution; otherwise, the Wilcoxon matched-pairs signed ranks sum test was used. Differences were considered statistically significant if the *P*

value was <0.05. All calculations were performed using SPSS software (version 24, SPSS, Inc.).

## 3. Results

In the present study, 52 eyes among 48 patients (35 male and 13 female) were evaluated. The characteristics of the study population are shown in Table 1. The mean age was 50.27 ± 20.08 (range: 6–81) years. The mean follow-up time was 79.7 ± 18.84 (range: 60–121) months.

### 3.1. Refractive and Visual Outcomes

The mean preoperative BCVA was 0.83 ± 0.69 logMAR; the mean postoperative BCVA was 0.50 ± 0.45 logMAR at the last follow-up (*p* < 0.05)

**Figure 2:**
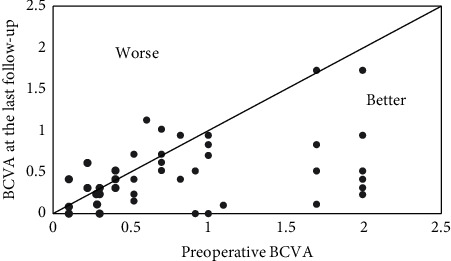
Scattergram of BCVA in 52 eyes that underwent SSF-IOL implantation. BCVA is represented in logMAR values. BCVA: best-corrected visual acuity; SSF-IOL: sutured scleral-fixated intraocular lens.

. In 44 eyes (84.62%), BCVA improved or remained unchanged; in 8 eyes (7.2%), it worsened (Figure 2). Reasons for BCVA decline included secondary epimacular membrane (3 eyes), progressive epimacular membrane (1 eye), optic atrophy (1 eye), and retinitis pigmentosa (3 eyes).

At the last follow-up visit, the mean SE was −1.00 ± 1.74 diopters, and the mean RPE was −0.67 ± 1.31 diopters. RPEs in 32 eyes (61.54%) were within 1.00 diopters and in 15 eyes (28.85%) were within 2.00 diopters. In 2 eyes (3.85%), RPE exceeded 4.00 diopters; both had high axial myopia (27.31 mm and 29.08 mm) and posterior scleral staphyloma, and their ALs had been measured by A-ultrasound in a county hospital, which might explain the unexpectedly large postoperative RPEs.

### 3.2. Complications

#### 3.2.1. Intraoperative

In 3 eyes (2 patients), vitreous hemorrhage occurred in association with passing the 10-0 polypropylene suture through the sclera 1.5 mm posterior to the limbus. In 1 eye, the hemorrhage appeared to arise from extraocular blood wicked into the eye through the puncture; it was mild and stopped after hemostasis. In the other 2 eyes, the patient had Marfan syndrome and took an oral anticoagulant (Warfarin) after cardiac surgery; the hemorrhage may have come from the ciliary body. Surgery proceeded and the vitreous hemorrhage resolved within 2 weeks. There was no other case of intraoperative complications such as choroidal detachment or suprachoroidal hemorrhage.

#### 3.2.2. Postoperative

**Figure 3:**
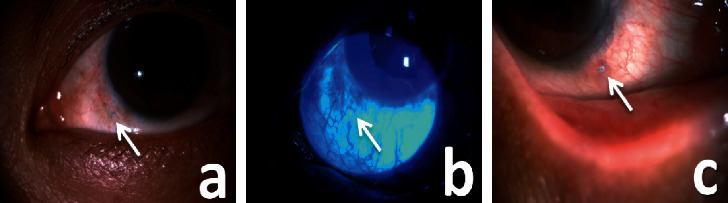
Slit-lamp microscopy images of the suture knot. (a) Suture knot visible under the conjunctiva. (b) Negative bulbar conjunctival fluoresce staining. (c) Suture exposure 5 years postsurgery.

**Figure 4:**
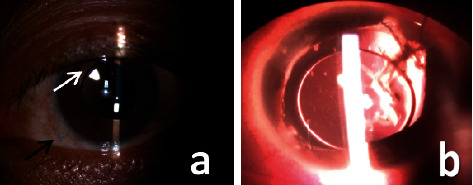
Slit-lamp microscopy images of well-centered IOLs. (a) Stable IOL position 97 months postsurgery. The black arrow points to the suture knot seen under conjunctiva. The white arrow points to the iris defect and pupil distortion due to previous trauma. (b) Well-placed IOL in a traumatic eye with atrophic iris 120 months postsurgery.

As shown in Table 2, early complications included transient elevated IOP in 5 eyes (9.62%) and hypotony in 1 eye (1.92%) that had previous PPV surgery. Late complications included retinal detachment, subconjunctival suture knot exposure, and persistent elevated IOP in 1 eye (1.92%) each; the latter occurred in an eye with GRAVES. Epimacular membrane occurred in 5 eyes (9.62%). In the eye with RD, the 1/5 PD round hole was located near the 9 : 30 o'clock 20G trocar position, away from the suture-fixation position. We observed few complications associated with sutures; in most patients, suture knots could be seen in the subconjunctiva (Figure 3(a)), but staining was negative (Figure 3(b)). Only in 1 eye (1.92%), one suture knot exposure occurred 5 years after surgery with positive staining, but no suture knot erosion was observed (Figure 3(c)). We performed a conjunctival separation and coverage to rescue it. No optical disturbing IOL tilt or decentration was observed (Figure 4).

It is notable that no suture breakage or IOL displacement was observed during follow-up of any patient in this study.

## 4. Discussion

Compared to iris-fixed IOL and AC-IOL, SF-IOL is superior in protecting the integrity of the anterior chamber, minimizing uveal contact, and independence of the presence of iris tissue [3, 8]. However, due to the need for vitrectomy equipment and specialized surgical skill, sutureless SF-IOL is not likely to be widely used in primary hospitals and eye centers, especially in undeveloped/developing countries. There are few reports of sutureless SF-IOL with extended follow-up [4, 9–11]. In contrast, the SSF-IOL is a time-tested method initially described in 1986 by Malbran et al. [34]. Its long-term track record and independence from vitrectomy equipment has made it a primary implant technique worldwide in patients without sufficient capsular support [14–24].

We report the long-term outcomes of SSF-IOL implantation via a small (≤3 mm) corneal incision using 10-0 polypropylene suture. Included are cases performed at the beginning of the learning phase and those performed in two primary hospitals. Short- and long-term complications were infrequent and clinical outcomes were favorable. The SSF foldable IOL technique is less traumatic as fewer manipulations inside the eyeball are needed: suture presetting, suture out-pulling, and IOL inserting, with puncture performed when the eyeball was intact and the other procedures were finished under a small (≤3 mm) incision. For eyes having secondary lens implantation without coexistent vitreoretinal disorders, 38 eyes (73.08%) at the tertiary hospital and 5 eyes (9.62%) at the primary county hospitals did not require vitrectomy. When there was minor vitreous incarceration in the pupil area/corneal incision, the vitreous can be excised using scissors and then using a miotic agent. The use of a thinner 10-0 polypropylene suture preset through the ciliary sulcus with ab externo technique and smaller suture puncture were associated with minimal vitreous fluid outflow and only minor change in IOP. Little disturbance of the intraocular environment helps maintain the integrity and stability of the eyeball, such that infusion or AC maintainer is not needed.

Short-term complications included vitreous hemorrhage (5.77%), transient elevated IOP (9.62%), and hypotony (1.92%). Mild transient intravitreal hemorrhage was observed in 3 eyes (2 patients); one of these patients had Marfan syndrome and the AE might be attributable to Warfarin after cardiac surgery. In these patients, hemorrhage resolved within 2 weeks. In another case, extraocular blood may have wicked into the eye through the puncture; however, it was mild and resolved with well hemostasis. The incidence of vitreous hemorrhage was comparable to previous reports by Yeung et al. [35] and Zhao et al. [24] (5% and 6.6%, respectively). Transient elevated IOP was observed within 3 days postoperatively in 5 eyes. All eyes were stabilized with topical medication within a few days postsurgery. The supposed reasons of ocular hypertension included postoperative inflammation, retained viscoelastic agents, and temporary dysfunction of the trabecular meshwork [25]. Our study implanted a foldable SSF-IOL through a small corneal incision, using only a small amount of viscoelastic agents with minimal manipulation and vitreous disturbance. Even in the 4 cases with previous ocular hypertension resulting from vitreous incarcerate in the pupil area, IOP returned to normal after excising vitreous and implanting IOL. The ocular hypertension rate in our study was significantly lower than those in SSF-IOL studies with methyl methacrylate (PMMA) material (range: 10%–44%) [16, 25–27] and comparable to others with foldable material (range: 7%–11.5%) [20, 36, 37]. Hypotony caused by incision leakage was observed in 1 eye post-PPV 1 day postsurgery. Thereafter, the main incision was sutured at the end of surgery to prevent postoperative hypotony, especially in vitrectomized eyes, and no further hypotony occurred. Consequently, the hypotony rate in this study is in stark contrast to studies (range: 4.3%–9.4%) [16, 24, 37].

IOL dislocation due to suture breakage was a late complication and is considered by surgeons to be the greatest challenge of this technique. The incidence of this complication, typically observed 3–5 years postprocedure [14–24], is estimated to be 0%–57.69% in Caucasians [23–28] and 0% to 4.65% in Asians and Africans [14, 19, 20, 29–31]. In our study, there was no suture breakage with a mean follow-up of 79.7 months. The role of pigment is unclear and requires further randomized, multicenter prospective study. Furthermore, according to some reports, the risk of postoperative suture breakage was greater in younger patients (range: 12%–24%) [17, 18]; however, in our study, there was no breakage in 13 patients who are <40 years old. An Indian report by Bhojwani et al. [31] including 12 children (under 16 years old) also found no suture breakage. Thus, we consider suture breakage in younger Asian patients is worth further investigation.

Although 9-0 polypropylene suture has been widely used recently [32, 38, 39], our study revealed no suture breakage, perhaps demonstrating the stability of 10-0 polypropylene scleral fixation sutures. This result was consistent with Luk et al. [14], with a mean follow-up of 73.4 months. We consider 10-0 polypropylene suture to have some advantages. First, a 10-0 polypropylene suture knot is smaller than a 9-0 knot and may produce fewer complications such as scleral atrophy above the knot and erosion of the stiff cutting ends [14, 26]. In most patients in our study, suture knots were seen in the subconjunctiva, but bulbar conjunctival fluorescein staining was negative (Figures 3(a) and 3(b)). Among 100 suture knots in 52 eyes, only 1 exposure was observed 5 years postsurgery with positive staining (Figure 3(c)), which was rescued by conjunctival separation and coverage. Smaller knots and softer thread arms of the 10-0 suture might produce less suture exposure and erosion. Second, when presetting sutures, the thicker and stiffer texture of a 9-0 or 8-0 suture may result in larger puncture allowing more intravitreous fluid leakage. What is more, there are several reports of a 10-0 polypropylene suture knotless *Z*-suture technique that demonstrate promising clinical results with follow-up from 6 to 135 months and that might further enhance SSF-IOL implantation [22, 23].

Other long-term AEs, including RD and significant lens tilt or decentration, were also infrequent in our study. RD was seen in 1 eye (1.92%) 3 years postsurgery with the retinal hole located away from the suture-fixation position (11 o'clock). We ascribe the detachment to the vitreous traction around the trocar rather than suture-fixation surgery. The retina was reattached successfully with no negative sequelae. Lens tilt and decentration are well-documented complications in SSF-IOL implantation. Durak et al. [40] reported a 16.7% rate of lens tilt or decentration. Lens tilt develops due to asymmetric suture placement, and decentration occurs due to the asymmetric attachment of suture haptics on the scleral bed, loose suture, suture breakage, or other causes [40]. We identified no significant IOL decentration or tilt in our study at a mean follow-up of 79.7 months.

After IOL implantation, all patients had improved vision. In 44 eyes (84.62%), BCVA improved or remained unchanged and worsened in 8 eyes (7.2%) at the last follow-up visit. Most previous studies showed increased or unchanged BCVA after SF-IOL implantation in 86.2%–92.8% of cases [24, 32, 35]. Visual outcomes in our study were impressive and comparable to the previous study with a high rate of BCVA improvement, demonstrating long-term stability of SSF foldable IOL. Those eyes with worsening BCVA over the follow-up period resulted from epimacular membrane, optic atrophy, and retinitis pigmentosa, which were inextricable. At the last follow-up, RPEs in 32 eyes (61.54%) were within 1.00 diopters and in 15 eyes (28.85%) were within 2.00 diopters. There is no consensus on the target spherical equivalent when implanting an SF-IOL. Because sulcus-fixated IOL is located more anteriorly than in-the-bag fixation, postoperative refraction can lead to a myopic shift from the predicted value. In our study, an unexpected myopic shift occurred in 2 eyes (−4.22 diopters and −5.74 diopters), both at county hospitals, with high axial myopia (27.31 mm and 29.08 mm) and posterior scleral staphyloma. Due to the lack of optical biometry there, ALs were measured by A-ultrasound, which could explain the large postoperative spherical equivalent deviation. Therefore, we strongly recommended taking careful repeated measures of AL and applying precise optical biometry in patients with high axial myopia and posterior scleral staphyloma.

There were some limitations to this study, primarily the retrospective design and the absence of a control group. However, its greatest strength was the long (>5 years) follow-up period. Another limitation was substantial (49%) loss to follow-up, which might introduce selection bias. Nevertheless, in our study, patient data were collected retrospectively for more than 5 years, and the loss to follow-up was a limitation inherent to the long-term retrospective design. Anterior segment optical coherence tomography or Scheimpflug images were not reported as a more accurate indication of IOL tilt or decentration; therefore, large, prospective, and long-term randomized clinical trials are required to further substantiate the therapeutic benefits demonstrated in this study.

## 5. Conclusions

Our study validated the beneficial long-term outcomes of SSF foldable IOL implantation in China via a small (≤3 mm) corneal incision using 10-0 polypropylene suture. This technology was safe, easy to master, and easily replicated. Both short- and long-term AEs (particularly suture-related complications) were rare and long-term visual outcomes were stable. Free from relying on complicated resources, such as expensive equipment and vitrectomy skills, SSF-IOL technology may be especially useful in primary hospitals and eye centers, and in underdeveloped or developing settings.

## Figures and Tables

**Table 1:** Patient characteristics.

Parameter	
Male : female	35 : 13
Age (years, mean ± SD)	50.27 ± 20.08
Follow-up time (months, mean ± SD)	79.7 ± 18.84
Right eye : left eye	27 : 25

Preoperative comorbidities, *n* (%)	

Trauma	12 (23.08%)
Postglaucoma surgery	2 (3.85%)
Glaucoma with vitreous incarceration	4 (7.69%)
History of retinal detachment	5 (9.62%)
Marfan syndrome	3 (5.77%)
Myopic degeneration	2 (3.85%)

Surgical indication, *n* (%)	

Aphakia after complicated cataract surgery	29 (55.77%)
Aphakia after traumatic cataract surgery	10 (19.23%)
Aphakia resulting from previous pars plana vitrectomy (PPV) for RD or trauma	4 (7.69%)
Dislocated crystalline lens	6 (11.54%)
Dislocated IOL	3 (5.77%)

**Table 2:** Distribution and management of postoperative complications in eyes undergoing SSF-IOL.

Complication				
Early (≤1 month)	*n* (%)	Mean duration postsurgery	Range	Management
Increased IOP	5 (9.62)	1.6 ± 0.8 days	1–3 days	Medical management
Transient vitreous hemorrhage	3 (5.77)	1 day	1 day	Medical management; resolved within 2 weeks
Transient hypotony	1 (1.92)	1 day	1 day	Suture the incision

Late (>1 month)	*n* (%)	—	—	—
Suture knot exposure	1 (1.92)	5 years	5 years	Surgical management
Retinal detachment	1 (1.92)	3 years	3 years	Surgical management
Increased IOP	1 (1.92)	6 years	6 years	Medical management

## Data Availability

The dataset used for analyses may be requested from the corresponding author for use in scholarly work related to the field.

## Supplementary Materials

Supplementary Materials The supplementary materials are two videos which describe the surgical procedure in detail.
