# Supplementary material for: Outcomes and Complications of Sutured Scleral-Fixated Foldable Intraocular Lens Implantation: A Retrospective Study of 5-Year Follow-Up
Source: J Ophthalmol. 2021 Jul 16;2021:5525064. doi: 10.1155/2021/5525064 (PMC8310451; doi:10.1155/2021/5525064)
Supplement: Supplementary Materials — The supplementary materials are two videos which describe the surgical procedure in detail. [file 5525064.f1.docx]

The three-piece IOL video showed the surgical procedures from the begining.

<https://drive.google.com/file/d/1Ff5wC1Uf1pqohpw1j-S3kiiTwfMbbjhp/view?usp=sharing>

 The one-piece IOL video showed the surgical procedures after presetting 10-0 polypropylene suture.

<https://drive.google.com/file/d/1uki4IiNJpYZih7cXuhU6UobP-NaIClqO/view?usp=sharing>
